# Perioperative predictors of complications in open abdominal aortic surgery: A retrospective analysis under a structured perioperative goal-directed therapy protocol

**DOI:** 10.1016/j.jatmed.2025.10.002

**Published:** 2025-12-02

**Authors:** Rosanna Carmela De Rosa, Antonio Romanelli

**Affiliations:** a Anesthesia and Intensive Care, AORN Ospedali dei Colli - "D. Cotugno" Hospital, 80131 Naples, Italy; b Anesthesia and Intensive Care, AOU “San Giovanni di Dio e Ruggi d′Aragona”, 84131 Salerno, Italy

**Keywords:** Abdominal aortic aneurysm, Hemodynamic monitoring, Perioperative goal-directed therapy, Postoperative complications, Risk factors, Fluid therapy

## Abstract

**Background:**

Perioperative goal-directed therapy (PGDT) optimizes hemodynamics in high-risk vascular surgery. However, data on postoperative risk factors and temporal hemodynamic and arterial blood gas (ABG) data patterns within PGDT-managed patients undergoing open elective abdominal aortic surgery (OEAAS) are limited.

**Methods:**

We conducted an explorative retrospective cohort study of patients undergoing sub-renal OEAAS managed with a structured PGDT protocol from the induction of anesthesia to six hours postoperatively. We collected clinical, hemodynamic, and ABG data at predefined perioperative time points. Patients were stratified by the occurrence of postoperative complications. Differences between groups were evaluated with appropriate tests. Univariate and bivariate Firth logistic regression analyses identified complication predictors, calculating odds ratio (OR) and 95 % confidence interval (95 % CI). Hemodynamic and ABG trends were assessed using the Aligned Rank Transform (ART) test. A p-value < 0.05 was significant.

**Results:**

Among 101 patients, postoperative complications occurred in 9.9 %, and the 30-day mortality rate was 4.0 %. Bivariate analysis identified longer surgery time (OR 1.01, 95 % CI 1.00–1.02, p = 0.034), higher postoperative fluid input (OR 1.67, 95 % CI 1.10–3.52, p = 0.016), and more positive fluid balance (OR 4.10, 95 % CI 1.53–16.76, p = 0.002) as associated with complications. ART indicated that patients with complications showed different trends in ScvO_2_ (p = 0.031) and lactate (p < 0.001).

**Conclusions:**

In patients managed with PGDT, postoperative complications were associated with surgical complexity, positive fluid balance, and markers of impaired oxygen delivery. Monitoring dynamic trends of ScvO₂ and lactate may help identify high-risk patients and guide individualized hemodynamic management.

## Introduction

Open elective abdominal aortic surgery (OEAAS) is a major surgical intervention that places significant physiological stress on patients, especially those with limited cardiovascular reserve. The pathophysiological burden of OEAAS is primarily driven by the profound hemodynamic fluctuations during critical surgical phases, particularly during aortic clamping and declamping. These maneuvers can abruptly change aortic impedance, preload, and myocardial contractility, resulting in unstable cardiac output and variability in oxygen delivery (DO_2_).^1^ Moreover, intraoperative and postoperative blood loss can decrease hemoglobin (Hb) concentration, further impairing tissue oxygenation. Collectively, these factors contribute to a 30-day mortality rate of 1–5 % in contemporary surgical series.^2^, ^3^, ^4^

Perioperative goal-directed therapy (PGDT) is an individualized strategy to optimize hemodynamic parameters and organ perfusion through dynamic monitoring, protocolized fluid, and vasopressor use. Clinical trials and meta-analyses have examined PGDT's efficacy in various surgical settings, showing improvements in morbidity and reductions in mortality among select high-risk populations.^5^, ^6^, ^7^, ^8^, ^9^, ^10^, ^11^ However, the generalizability of these findings to vascular surgery is limited. The literature on open aortic surgery is heterogeneous, with significant variations in patient populations, monitoring tools, and therapeutic targets.^12^, ^13^, ^14^, ^15^, ^16^

Most studies compare PGDT to standard care, but evidence on risk factors for postoperative complications and the evolution of physiological markers in PGDT cohorts is limited.

This exploratory retrospective cohort study aimed to investigate the associations between clinical, hemodynamic, and metabolic variables and postoperative complications in patients undergoing OEAAS under a standardized PGDT protocol. We also analyzed temporal trends in hemodynamic and arterial blood gas (ABG) parameters to identify patterns linked to adverse outcomes.

## Materials and methods

### Patient enrollment

This is a retrospective analysis of a prospectively collected cohort from the VASCO (VAscular Surgery Cardiac Output Optimization) study, a multicenter protocol investigating hemodynamic patterns and outcomes in patients undergoing vascular surgery managed with PGDT. For this analysis, we included only patients who underwent sub-renal OEAAS and were managed perioperatively with a structured PGDT protocol at AORN dei Colli Monaldi Hospital (Naples, Italy) between April 2016 and August 2020.

During the anesthesiologist consultation, age, gender, weight, height, body mass index (BMI), American Society of Anesthesiology (ASA) physical status and comorbidities [coronary artery disease (CAD), previous coronary aortic bypass graft (CABG) or percutaneous transluminal coronary angioplasty (PTCA)], cardiomyopathy, arterial hypertension, diabetes mellitus, obesity (defined as BMI ≥ 30 kg m^−2^), chronic kidney disease (CKD), chronic obstructive pulmonary disease (COPD), transient ischemic attack (TIA) or stroke were collected. The Vascular-Physiological and Operative Severity Score for the enUmeration of Mortality and Morbidity (V-POSSUM) was calculated for each patient to provide a more accurate preoperative risk stratification.^17^

Exclusion criteria defined a priori in the VASCO protocol included: patients aged ≥ 85 years, ASA physical status ≥ IV, NYHA class ≥ III, and V-POSSUM predicted morbidity < 15 %. This threshold aimed to include moderate-to-high-risk patients, best suited for PGDT. Additionally, we added an exclusion criterion for this center-specific analysis: patients with contraindications to intraoperative autologous blood salvage (e.g., active infection, risk of tumor cell dissemination). This was because the absence of blood salvage would necessitate alternative transfusion and fluid management strategies, potentially altering hemodynamic trends and outcomes. Moreover, many conditions contraindicating blood salvage are linked to poor prognosis, representing confounding factors for clinical outcomes.

All data were fully anonymized before analysis in accordance with Italian privacy legislation (Legislative Decree 196/2003, as amended by Legislative Decree 101/2018). Information was entered into a dedicated electronic database (Microsoft Excel 2016, Microsoft Corp., Redmond, WA, USA). The study was conducted following the ethical principles of the Declaration of Helsinki.

### Data collection

A structured PGDT protocol, based on stroke volume index (SVI) optimization,^18^, ^19^ was implemented for all patients, beginning immediately after anesthesia induction and continuing for at least six hours postoperatively in the Postoperative Care Unit (POCU). For details, see Supplementary.

The following intraoperative and postoperative data were recorded: (1) Timing variables: duration of anesthesia, surgery, and aortic clamping; (2) Fluid management: total intraoperative and postoperative fluid input, including crystalloids, colloids, autologous blood salvage, and transfused blood products [packed red blood cells (PRBC) and fresh frozen plasma (FFP)]; (3) Fluid output: estimated blood loss and urine output during both intraoperative and early postoperative periods; (4) Fluid balance: calculated as the difference between total input and output volumes; (5) Norepinephrine (NE) bolus and diuretic (furosemide) administration.

All volumes were standardized by body weight and anesthesia time for intraoperative values and by weight and duration of observation for postoperative values. Blood loss and salvaged blood volumes were standardized per kilogram of body weight and surgical time.

Hemodynamic and arterial blood gas (ABG) parameters were recorded at predefined time points: T_0_: immediately after the induction of general anesthesia (start of PGDT protocol); T_1_: before aortic clamping; T_2_: after aortic declamping; T_3_: end of surgery; T_4_: two hours after admission to the POCU; T_5_: six hours after POCU admission (end of PGDT protocol).

At each time point, the following variables were recorded: MAP, CVP, heart rate (HR), SVI, cardiac index (CI), ScvO_2_, SpO_2_, and ABG parameters [pH, base excess (BE), lactate, and Hb].

Postoperative complications were documented and categorized according to the affected organ system: (1) Respiratory complications: prolonged mechanical ventilation (>12 h), postoperative respiratory failure, pneumonia, reintubation; (2) Cardiovascular complications: hypotension requiring pharmacologic support, new-onset arrhythmia, myocardial ischemia, cardiac arrest with return of spontaneous circulation (ROSC); (3) Renal complications: acute kidney injury defined according to RIFLE/AKIN criteria^20^; (4) Other complications: postoperative bleeding, stroke, wound infection, mesenteric ischemia, etc.

Complications and deaths occurring within 30 days postoperatively were considered for analysis.

### Statistical analysis

Given the retrospective and explorative nature of the present analysis, no a priori sample size calculation was performed. In case of missing data, analyses were performed using available-case data. No imputation methods were applied.

We used descriptive statistics to summarize the data. Categorical variables were reported as frequencies and percentages. Continuous variables were assessed for normality with a density plot. Normally distributed variables were expressed as mean and standard deviation (SD), otherwise as median and quartiles (Q_1_–Q_3_), along with minimum and maximum values.

Patients were divided into two groups based on the occurrence of postoperative complications. Depending on the data distribution, continuous variables were tested with the Student’s (or Welch’s) or Mann-Whitney test. Categorical variable differences were analyzed using the χ^2^ test with Yates’ continuity correction.

Univariate and bivariate logistic regression analyses assessed the relationship between independent variables and postoperative complications. With a low event rate, we applied Firth’s penalized likelihood logistic regression to minimize small-sample bias and enhance coefficient stability.^21^ Results are reported as odds ratios (OR) with 95 % confidence intervals (95 % CI). In the bivariate model, OR was adjusted for the V-POSSUM morbidity score, selected a priori as a validated composite of baseline surgical risk that parsimoniously summarizes comorbidity burden and operative complexity. Variables with frequencies below five were excluded from the analysis.

To evaluate hemodynamic and ABG trends over time (T_0_–T_5_), we conducted a two-way repeated-measures analysis of variance (ANOVA) or Aligned Rank Transform (ART) test according to data distribution. We analyzed both absolute values and percentage changes (considering T_0_ as reference). When significant time-group interactions were detected, pairwise post hoc comparisons were conducted using appropriate tests, adjusting p-values with the False Discovery Rate (FDR) correction for multiple testing.

All tests were performed with an α = 0.05, and a *p*-value < 0.05 was considered statistically significant. Graphical representations and summary tables were created to illustrate key findings.

All statistical analyses were conducted using R Studio (Posit Software, PBC, Boston, MA, USA).

## Results

### Population characteristics

Between April 2016 and August 2020, 104 patients were scheduled for sub-renal OEAAS. In three cases, intraoperative autologous blood salvage was not performed due to clinical contraindications (two patients with active malignancy and one with sepsis). No missing data were present for the variables included in the analysis. The final analysis included 101 patients (Table 1).

**Table 1:** Baseline population characteristics and group comparison stratified by postoperative complications.

**Variable**		**Overall**		**Group Comparison**		
		**Result**	**Min-Max**	**No-Complications (n = 91)**	**Complications (n = 10)**	***p*****-value**
Sex, male (%)		91 (90.1 %)	-	83 (91.2 %)	8 (80.0 %)	0.569
Age (years)		69.0 (64.0–75.0)	43.0–82.0	69.0 (64.0–74.0)	70.5 (62.0–76.0)	< 0.001
BMI (kg m^−2^)		25.7 (23.7–28.4)	19.0–37.0	25.7 (23.7–28.9)	26.0 (23.2–27.7)	< 0.001
ASA physical Status						
	II (%)	6 (5.9 %)	-	5 (5.5 %)	1 (10.0 %)	1.000
	III (%)	95 (94.1 %)	-	86 (94.5 %)	9 (90.0 %)	
**Comorbidities**						
Arterial hypertension (%)		84 (83.2 %)	-	77 (84.6 %)	7 (70.0 %)	0.467
COPD (%)		49 (48.5 %)	-	43 (47.2 %)	6 (60.0 %)	0.665
CAD (%)		43 (42.6 %)	-	37 (40.6 %)	6 (60.0 %)	0.402
CABG/PTCA (%)		30 (29.7 %)	-	27 (29.7 %)	3 (30.0 %)	1.000
Diabetes (%)		19 (18.8 %)	-	18 (19.8 %)	1 (10.0 %)	0.745
Cardiomiopathy (%)		18 (17.8 %)	-	16 (17.6 %)	2 (20.0 %)	1.000
Obesity (%)		14 (13.9 %)	-	14 (15.4 %)	0 (0.0 %)	0.393
CKD (%)		12 (11.9 %)	-	10 (11.0 %)	2 (20.0 %)	0.748
TIA/Stroke (%)		11 (10.9 %)	-	11 (12.1 %)	0 (0.0 %)	0.529
V-POSSUM morbidity (%)		34.8 (28.7–42.3)	15.3–90.5	34.8 (28.7–42.3)	40.7 (34.8–59.4)	< 0.001
V-Possum mortality (%)		2.2 (1.7–2.7)	0.6–24.7	2.2 (1.7–2.7)	2.4 (2.0–4.3)	< 0.001
**Procedural Times**						
Anestesia time (min)		270.0 (210.0–300.0)	180.0–510.0	270.0 (210.0–300.0)	270.0 (270.0–322.5)	< 0.001
Surgery time (min)		240.0 (180.0–270.0)	150.0–480.0	240.0 (180.0–270.0)	240.0 (240.0–292.5)	< 0.001
Aortic clamping (min)		60.0 (40.0–60.0)	30.0–90.0	55.0 (40.0–60.0)	60.0 (41.2–63.7)	< 0.001
**Intraoperative fluid management and balance**						
Crystalloid (mL kg^−1^ h^−1^)*		2.0 ± 0.6	0.9–4.5	2.0 ± 0.6	2.1 ± 0.8	0.702
Colloid (mL kg^−1^ h^−1^)*		2.5 ± 0.9	0.8–5.1	2.5 ± 0.8	2.3 ± 0.7	0.578
Colloid challenge						
	1–2 (%)	31 (30.7 %)	-	29 (31.9 %)	2 (20.0 %)	0.716
	3–4 (%)	63 (62.4 %)	-	56 (61.5 %)	7 (70.0 %)	
	> 4 (%)	7 (6.9 %)	-	6 (6.6 %)	1 (10.0 %)	
Noradrenaline bolus						
	none (%)	55 (54.4 %)	-	46 (50.5 %)	9 (90.0 %)	0.050
	1–3 (%)	27 (26.7 %)	-	27 (29.7 %)	0 (0.0 %)	
	> 3 (%)	19 (18.8 %)	-	19 (19.8 %)	1 (10.0 %)	
Volume blood savage (mL kg^−1^ h^−1^)*		2.2 ± 1.0	0.5–4.6	2.2 ± 1.0	2.5 ± 0.9	0.415
Blood loss (mL kg^−1^ h^−1^)*		3.9 ± 1.6	1.3–7.6	3.8 ± 1.5	4.7 ± 1.7	0.159
PRBC (%)		14 (13.9 %)	-	12 (13.2 %)	2 (20.0 %)	0.913
FFP (%)		3 (3.0 %)	-	2 (2.2 %)	1 (10.0 %)	0.690
Urine output (mL kg^−1^ h^−1^)		1.7 (1.3–2.3)	0.7–4.7	1.7 (1.3–2.3)	1.2 (1.2–2.9)	< 0.001
Diuretic (%)		29 (28.7 %)	-	25 (27.5 %)	4 (40.0 %)	0.643
Fluid input (mL kg^−1^ h^−1^)		6.8 (5.8–8.4)	4.1–13.8	6.7 (5.8–8.4)	8.0 (5.9–9.0)	< 0.001
Fluid output (mL kg^−1^ h^−1^)		6.0 (5.0–7.2)	3.6–11.2	5.9 (5.3–7.1)	7.1 (5.3–7.9)	< 0.001
Fluid balance (mL kg^−1^ h^−1^)		+ 0.9 ± 1.0	−1.7–+ 4.1	+ 1.1 ± 1.0	+ 0.7 ± 0.5	0.099
**Postoperative fluid management and balance**						
Crystalloid (mL kg^−1^ h^−1^)*		2.1 ± 0.6	0.3–3.6	2.1 ± 0.6	2.4 ± 0.5	0.081
Colloid (mL kg^−1^ h^−1^)		0.5 (0.0–0.8)	0.0–3.3	0.5 (0.0–0.8)	0.5 (0.1–1.1)	< 0.001
Colloid challenge						
	none (%)	27 (26.7 %)	-	24 (26.4 %)	3 (30.0 %)	0.120
	1–2 (%)	68 (67.3 %)	-	63 (69.2 %)	5 (50.0 %)	
	> 2 (%)	6 (5.9 %)	-	4 (4.4 %)	2 (20.0 %)	
PRBC (%)		3 (3.0 %)	-	2 (2.2 %)	1 (10.0 %)	0.690
FFP (%)		1 (1.0 %)	-	0 (0.0 %)	1 (10.0 %)	0.177
Urine output (mL kg^−1^ h^−1^)		1.5 (1.3–2.0)	0.6–4.0	1.5 (1.3–2.0)	1.3 (1.2–1.8)	< 0.001
Fluid input (mL kg^−1^ h^−1^)		2.8 (2.5–3.1)	1.3–10.2	2.8 (2.5–3.1)	3.2 (2.7–3.6)	< 0.001
Fluid output (mL kg^−1^ h^−1^)		2.3 (2.0–2.7)	1.3–6.0	2.3 (2.1–2.7)	2.3 (2.1–2.8)	< 0.001
Fluid balance (mL kg^−1^ h^−1^)*		+ 0.5 ± 0.7	−1.6–4.2	+ 0.4 ± 0.6	+ 1.2 ± 1.1	0.059

Continuous variables are expressed as median (Q_1_–Q_3_) or mean±standard deviation (SD), depending on distribution, along with minimum–maximum values. Categorical variables are presented as absolute numbers and percentages. Differences were tested with Student (or Welch Test*) or Mann-Whitney test for continuous variables according to data distribution. For categorical variables, differences were analyzed with χ^2^ test with Yates’ continuity correction. A p < 0.05 was considered significant. BMI, Body Mass Index; ASA, American Society of Anesthesiologists; COPD, Chronic Obstructive Pulmonary Disease; CAD, Coronary Artery Disease; CABG, Coronary Artery Bypass Grafting; PTCA, Percutaneous Transluminal Coronary Angioplasty; CKD, Chronic Kidney Disease; TIA, Transient Ischemic Attack; V-POSSUM, Vascular-Physiological and Operative Severity Score for the enUmeration of Mortality and Morbidity; PRBC, Packed Red Blood Cells; FFP, Fresh Frozen Plasma.

Briefly, 91 patients (90.1 %) were male, with a median age of 69.0 years (Q_1_–Q_3,_ 64.0–75.0). Most patients were classified as ASA III (n = 95, 94.1 %), while the remainder were ASA II (n = 6, 5.9 %). Common comorbidities included arterial hypertension (n = 84, 83.2 %), COPD (n = 49, 48.5 %), and CAD (n = 43, 42.6 %). The median predicted morbidity and mortality rates according to the V-POSSUM score were 34.8 % (Q_1_–Q_3_, 28.7–42.3 %) and 2.2 % (Q_1_–Q_3_, 1.7–2.7 %), respectively. The median surgery duration was 240.0 min (Q_1_–Q_3_, 180.0–270.0), with a median aortic clamping time of 60.0 min (Q_1_–Q_3_, 40.0–60.0) and a median anesthesia time of 270.0 min (Q_1_–Q_3_, 210.0–300.0).

### PGDT and fluid balances

During the intraoperative period, 31 patients (30.7 %) required up to two colloid challenges, 63 (62.4 %) for up to four, and seven patients (6.9 %) received more than four fluid challenges. To maintain MAP≥ 65 mmHg after SVI optimization, 27 patients (26.7 %) received up to three NE boluses, while 19 (18.8 %) required more than three boluses.

The mean volume of autologous salvaged blood reinfused intraoperatively was 2.2 mL kg^−1^ h^−1^ (SD 1.0), while the median estimated blood loss was 3.9 mL kg^−1^ h^−1^ (SD 1.6). Fourteen patients (13.9 %) required intraoperative PRBC transfusions, and three received FFP (3.0 %). Twenty-nine patients (28.7 %) received furosemide intraoperatively. The median urine output was 1.7 mL kg^−1^ h^−1^ (Q_1_–Q_3_, 0.7–4.7). The mean intraoperative fluid balance was + 0.9 mL kg^−1^ h^−1^ (SD 1.0).

In the postoperative period, 27 (26.7 %) patients did not require colloid fluid challenge, while 65 (64.3 %) required up to two. Six patients (5.9 %) required more than two postoperative fluid challenges. Postoperative transfusions were limited to 3 patients (3.0 %) for PRBC and one patient (1.0 %) for FFP. The median urine output was 1.5 mL kg^−1^ h^−1^ (Q_1_–Q_3_, 1.3–2.0). The mean postoperative fluid balance was + 0.5 mL kg^−1^ h^−1^ (SD 0.7).

### Postoperative complications and mortality rates

Ten patients (9.9 %) had postoperative complications, detailed in Table S1. The most common were acute kidney injury (n = 6, 5.9 %) and reintubation (n = 5, 5.0 %).

No intraoperative deaths occurred. Four patients (4.0 %) died within 30 days, including one from sudden ventricular fibrillation on postoperative day 3 in the POCU. The other three deaths resulted from surgical complications: one due to postoperative bleeding and two from mesenteric ischemia.

### Statistical comparison between the complications and the no-complications groups

Table 1 presents the comparative analysis between patients who developed postoperative complications (n = 10) and those who did not (n = 91). The 30-day mortality rate was significantly higher in the complication group (40.0 %) compared to 0.0 % in the no-complication group (p < 0.001).

Patients with postoperative complications were older (median 70.5 vs. 69.0 years, p < 0.001) and had a higher BMI (median 26.0 vs. 25.7 kg m^−2^, p < 0.001). They also had a worse preoperative risk profile, indicated by elevated V-POSSUM predicted morbidity (40.7 % vs. 34.8 % in no-complication patients, p < 0.001) and mortality risk (2.4 % vs. 2.2 %, p < 0.001).

A clear pattern emerged when comparing the durations of surgery and anesthesia. The median surgical (240.0 min) and anesthesia times (270.0 min) were the same in both groups, but the distribution was asymmetrical. In the complication group, procedures clustered in the upper range of both durations. Complication groups had longer aortic clamping time (60.0 vs. 55.0 min, p < 0.001).

Intraoperative fluid management revealed that the complication group had higher standardized fluid input (8.0 vs. 6.7 mL kg^−1^ h^−1^, p < 0.001) and output (7.1 vs. 5.9 mL kg^−1^ h^−1^, p < 0.001), but lower urine output (1.2 vs. 1.7 mL kg^−1^ h^−1^, p < 0.001). Notably, NE requirements differed (90.0 % in complication vs. 50.5 % in the no complication group, p = 0.05). No difference was observed in intraoperative fluid balance (p = 0.099).

Regarding postoperative fluid management, the complication group showed a lower median for urine output (1.3 vs. 1.5 mL kg^−1^ h^−1^ in the no-complication group, p < 0.001) and a higher median for fluid input (3.2 vs. 2.8 mL kg^−1^ h^−1^ in the no-complication group, p < 0.001). Despite equal median values, volumes for colloid and fluid output in the complication group were clustered in the upper range of the distribution (p < 0.001). No difference was noted in postoperative fluid balance (p = 0.059).

### Logistic regression analysis

The results of the univariate and bivariate logistic regression analyses are presented in Table 2.

**Table 2:** Univariate and adjusted logistic regression models for predictors of postoperative complications.

**Variable**		**Univariate Model**		**V-Possum Morbidity Model**	
		**OR (95 % CI)**	**P-value**	**OR (95 % CI)**	**P-value**
Sex, male		0.35 (0.08–2.05)	0.217	0.29 (0.06–1.76)	0.161
Age		1.02 (0.94–1.12)	0.629	0.98 (0.90–1.08)	0.756
BMI		0.94 (0.77–1.12)	0.514	0.97 (0.79–1.16)	0.765
ASA physical status					
	II	ref.	0.389	ref.	0.125
	III	0.40 (0.07–4.22)		0.15 (0.02–1.87)	
**Comorbidities**					
Arterial hypertension		0.40 (0.10–1.81)	0.217	0.37 (0.09–1.72)	0.191
COPD		1.61 (0.46–6.17)	0.458	1.46 (0.40–5.66)	0.564
CAD		2.10 (0.59–8.06)	0.248	1.55 (0.39–6.29)	0.526
CABG/PTCA		1.09 (0.25–3.99)	0.896	0.88 (0.19–3.33)	0.857
Diabetes		0.63 (0.06–3.00)	0.594	0.65 (0.06–3.19)	0.626
Cardiomiopathy		1.34 (0.24–5.47)	0.705	1.13 (0.19–4.74)	0.876
Obesity		0.25 (0.00–2.18)	0.260	0.30 (0.00–2.67)	0.339
CKD		2.28 (0.39–9.81)	0.323	2.06 (0.330–9.354)	0.401
TIA/Stroke		0.33 (0.00–2.92)	0.388	0.446 (0.00–4.17)	0.548
V-POSSUM morbidity		48.02 (1.01–2247.20)	0.049	-	-
V-POSSUM mortality		5797.15 (0.00–-)	0.224	-	-
**Procedural Times**					
Surgery time		1.01 (1.00–1.02)	0.047	1.01 (1.00–1.02)	0.034
Aortic clamping		1.01 (0.96–1.05)	0.665	1.02 (0.97–1.06)	0.496
**Intraoperative fluid management and balance**					
Crystalloid		1.35 (0.47–3.31)	0.548	1.29 (0.42–3.32)	0.626
Colloid		0.84 (0.37–1.76)	0.666	0.83 (0.35–1.78)	0.643
Colloid challenge					
	1–2	ref.	0.666	ref.	0.190
	3–4	1.57 (0.39–8.80)		1.66 (0.40–9.65)	
	> 4	2.72 (0.22–24.36)		3.34 (0.26–32.94)	
Noradrenaline bolus					
	none	ref.	0.059	ref.	0.049
	1–3	0.09 (0.00–0.75)		0.12 (0.00–1.06)	
	> 3	0.40 (0.04–1.92)		0.38 (0.04–1.88)	
Volume blood savage		1.32 (0.69–2.45)	0.389	1.18 (0.61–2.21)	0.608
Blood loss		1.38 (0.93–2.07)	0.105	1.29 (0.86–1.95)	0.218
PRBC		1.87 (0.32–7.84)	0.442	0.78 (0.09–4.49)	0.795
Urine output		1.38 (0.62–2.79)	0.397	1.26 (0.57–2.57)	0.537
Diuretic		1.00 (1.00–1.01)	0.111	1.51 (0.37–5.54)	0.547
Fluid input		1.17 (0.86–1.55)	0.294	1.08 (0.79–1.44)	0.621
Fluid output		1.33 (0.95–1.85)	0.093	1.24 (0.88–1.73)	0.211
Fluid balance		0.73 (0.33–1.41)	0.370	0.65 (0.29–1.27)	0.223
**Postoperative fluid management and balance**					
Crystalloid		2.84 (0.93–9.31)	0.068	2.70 (0.85–9.14)	0.093
Colloid		2.24 (0.85–6.05)	0.097	2.13 (0.81–5.63)	0.117
Colloid challenge					
	none	ref.	0.155	ref.	0.073
	1–2	0.61 (0.15–2.79)		0.64 (0.15–2.99)	
	> 2	3.89 (0.52–27.28)		3.51 (0.46–25.50)	
Urine output		0.48 (0.10–1.47)	0.230	0.48 (0.11–1.39)	0.196
Fluid input		1.73 (1.12–3.72)	0.012	1.67 (1.10–3.52)	0.016
Fluid output		1.49 (0.74–2.74)	0.237	1.41 (0.69–2.62)	0.310
Fluid Balance		3.61 (1.49–13.21)	0.003	4.10 (1.53–16.76)	0.002

The table reports odds ratios (OR), 95 % confidence intervals (95 % CI), and p-values from both univariate Firth logistic regression models and a multivariable model adjusted for V-POSSUM morbidity. BMI, Body Mass Index; ASA, American Society of Anesthesiologists; COPD, Chronic Obstructive Pulmonary Disease; CAD, Coronary Artery Disease; CABG, Coronary Artery Bypass Grafting; PTCA, Percutaneous Transluminal Coronary Angioplasty; CKD, Chronic Kidney Disease; TIA, Transient Ischemic Attack; V-POSSUM, Vascular-Physiological and Operative Severity Score for the enUmeration of Mortality and Morbidity; PRBC, Packed Red Blood Cells.

**Fig. 1:**
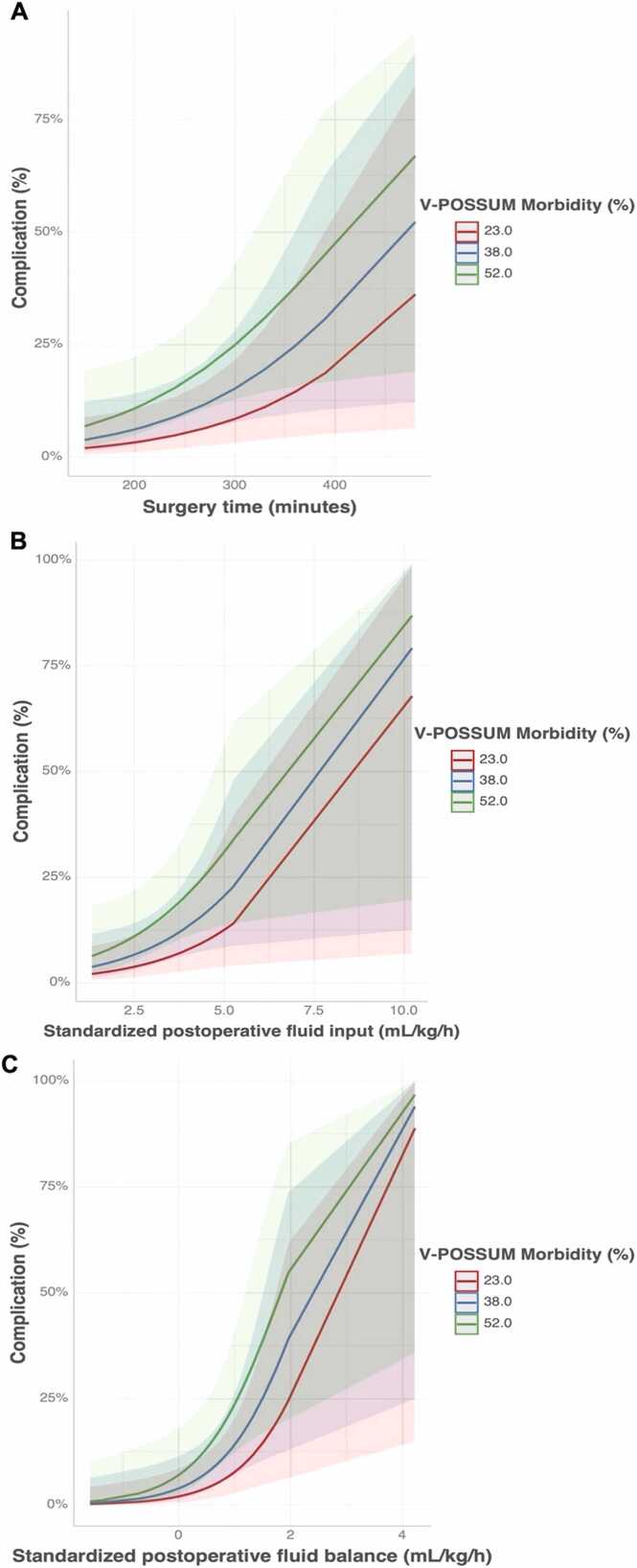
Marginal predicted probabilities of postoperative complications. (A)The likelihood of complications increases with longer surgical duration across all levels of predicted morbidity. (B) Higher standardized postoperative fluid input (mL kg^−1^ h^−1^) was associated with an increased risk of complications, particularly at higher morbidity scores. (C) A more positive standardized postoperative fluid balance was strongly associated with complications. Shaded areas represent 95 % confidence intervals.

In the univariate analysis, the following variables were significantly associated with the development of postoperative complications: V-POSSUM morbidity score (OR 48.02, 95 % CI 1.01–2247.20, p = 0.049), surgery time (OR 1.01, 95 % CI 1.00–1.02, p = 0.047), postoperative fluid input (OR 1.73, 95 % CI 1.12–3.72, p = 0.012), and postoperative fluid balance (OR 3.61, 95 % CI 1.49–13.21, p = 0.003). In the bivariate analysis, with adjustment for the V-POSSUM morbidity score, all the above variables remained statistically significant (Fig. 1).

In univariate analysis, administration of 1–3 NE boluses was associated with a lower odds of complications (OR 0.09, 95 % CI 0.00–0.75), though the p-value was slightly above the conventional significance threshold (p = 0.059). However, in the bivariate model, the association reached statistical significance (OR 0.12, 95 % CI 0.00–1.06) with a global p-value of 0.049. The administration of more than three NE boluses was associated with a nonsignificant reduction in complication risk in both univariate and bivariate models.

### Trends analysis

Table S2 reports the hemodynamic and ABG analysis parameters noted from T_0_ to T_5_ as absolute values and percentage changes relative to baseline (T_0_). Supplementary provides a complete description of the analytical approach and detailed statistical output.

ART test showed significant time-complication interaction for SVI (p = 0.010), SVI_Var_ (p = 0.005), ScvO_2Var_ (p = 0.031), SpO_2_ (p < 0.001), SpO_2Var_ (p < 0.001), lactate (p < 0.001), Lat_Var_ (p < 0.001), Hb (p = 0.026), and Hb_Var_ (p = 0.003).

**Fig. 2:**
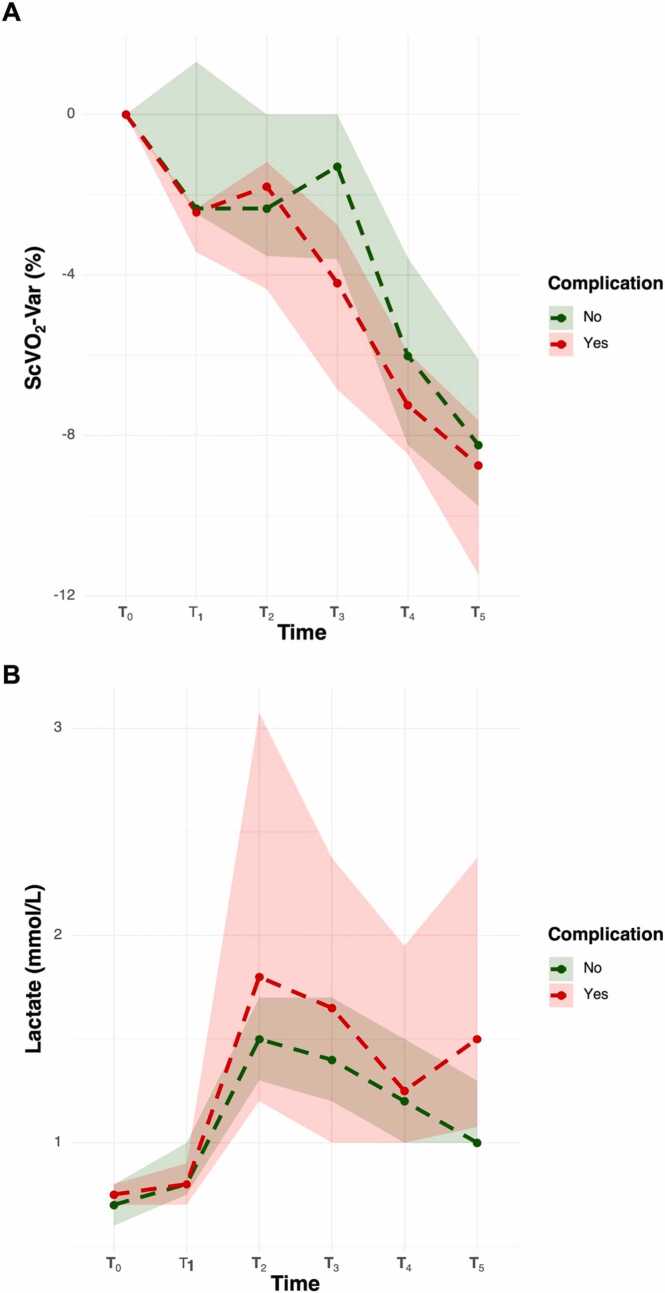
Trends of ScvO₂ variation and blood lactate concentration in patients with and without postoperative complications. (A) Percentage change in ScvO₂ (ScvO₂-var) from baseline (T₀) to 6 h postoperatively (T₅). Patients who developed complications showed greater and more persistent decreases in ScvO₂ across time points, particularly during aortic clamping (T₁) and after surgery (T₃–T₅), compared to those without complications. (B) Blood lactate concentrations increased more markedly and remained elevated longer in patients with complications, showing a declining trend in the non-complication group. Shaded areas represent 95 % confidence intervals. T_0_: immediately after the induction of general anesthesia (start of PGDT protocol); T_1_: before aortic clamping; T_2_: after aortic declamping; T_3_: end of surgery; T_4_: two hours after admission to the POCU; T_5_: six hours after POCU admission (end of PGDT protocol).

Pairwise comparisons (Table 3) revealed that patients who developed complications exhibited significantly greater reductions in ScvO_2Var_ at T_1_ (p = 0.047) and T_3_ (p = 0.040) compared to those without complications (Fig. 2A). At T_5_, serum lactate levels were significantly higher in the complication group (p = 0.049) (Fig. 2B). No other time points showed statistically significant between-group differences for the remaining parameters.

**Table 3:** Time course of hemodynamic, metabolic, and oxygenation parameters in patients with and without postoperative complications.

**Variable**	**Time**	**No-complication**	**Complication**	**P-value**
SVI (mL min^−1^ m^−2^)				
	T_0_	35.0 (32.0–40.0)	35.0 (30.0–36.8)	0.405
	T_1_	42.0 (38.0–45.5)	38.0 (37.2–41.5)	0.474
	T_2_	38.0 (35.0–42.0)	40.0 (38.2–42.0)	0.355
	T_3_	42.0 (40.0–45.0)	40.0 (37.2–43.5)	0.468
	T_4_	40.0 (38.0–43.0)	38.5 (36.0–42.0)	0.349
	T_5_	41.0 (39.0–45.0)	40.0 (35.8–40.0)	0.353
SVI_Var_ (%)				
	T_0_	0.0 (0.0–0.0)	0.0 (0.0–0.0)	-
	T_1_	+17.6 (+10.3–+25.0)	+22.5 (+11.4–+27.0)	0.622
	T_2_	+5.6 (-2.4–+13.2)	+13.0 (+9.3–+33.0)	0.059
	T_3_	+17.1 (+8.9–+26.5)	+20.0 (+7.1–+26.4)	0.772
	T_4_	+12.9 (+5.7–+20.0)	+18.3 (+5.9–+23.8)	1.000
	T_5_	+16.7 (+8.7–+23.5)	+11.0 (+6.2–+22.3)	0.732
ScvO_2_ (%)				
	T_0_	83.0 (80.0–85.0)	83.5 (80.5–85.0)	0.894
	T_1_	82.0 (78.0–83.0)	80.0 (78.5–82.8)	0.679
	T_2_	80.0 (78.0–83.0)	80.0 (79.2–82.5)	0.991
	T_3_	80.0 (79.5–82.0)	78.0 (78.0–80.5)	0.276
	T_4_	77.0 (75.0–79.0)	77.0 (75.2–77.8)	0.564
	T_5_	75.0 (74.0–77.0)	75.0 (73.5–75.8)	0.852
ScvO_2Var_ (%)				
	T_0_	0.0 (0.0–0.0)	0.0 (0.0–0.0)	-
	T_1_	−2.3 (−2.5–+1.3)	−2.4 (−3.4–−2.3)	0.047
	T_2_	−2.3 (−3.5–0.0)	−1.8 (−4.4–−1.2)	0.585
	T_3_	−1.3 (−3.6–0.0)	−4.2 (−6.9–−2.8)	0.040
	T_4_	−6.0 (−8.2–−3.6)	−7.2 (−8.5–−5.9)	0.237
	T_5_	−8.2 (−9.8–−6.1)	−8.7 (−11.5–−7.6)	0.229
SpO_2_ (%)				
	T_0_	100.0 (100.0–100.0)	100.0 (100.0–100.0)	1.000
	T_1_	100.0 (100.0–100.0)	100.0 (100.0–100.0)	1.000
	T_2_	100.0 (100.0–100.0)	100.0 (100.0–100.0)	1.000
	T_3_	100.0 (100.0–100.0)	100.0 (100.0–100.0)	1.000
	T_4_	99.0 (98.5–100.0)	99.0 (97.5–100.0)	1.000
	T_5_	98.0 (97.0–98.0)	97.5 (97.0–98.0)	1.000
SpO_2Var_ (%)				
	T_0_	0.0 (0.0–0.0)	0.0 (0.0–0.0)	-
	T_1_	0.0 (0.0–0.0)	0.0 (0.0–0.0)	1.000
	T_2_	0.0 (0.0–0.0)	0.0 (0.0–0.0)	1.000
	T_3_	0.0 (0.0–0.0)	0.0 (0.0–0.0)	1.000
	T_4_	−1.0 (−1.0–0.0)	−1.0 (−2.5–0.0)	1.000
	T_5_	−2.0 (−3.0–−2.0)	−2.5 (−3.0–−2.0)	1.000
Lactate (mmol L^−1^)				
	T_0_	0.7 (0.6–0.8)	0.7 (0.7–0.8)	0.823
	T_1_	0.8 (0.7–1.0)	0.8 (0.7–0.9)	0.933
	T_2_	1.5 (1.3–1.7)	1.8 (1.2–3.1)	1.000
	T_3_	1.4 (1.2–1.7)	1.6 (1.0–2.4)	1.000
	T_4_	1.2 (1.0–1.5)	1.2 (1.0–1.9)	0.763
	T_5_	1.0 (1.0–1.3)	1.5 (1.1–2.4)	0.049
Lat_Var_ (%)				
	T_0_	0.0 (0.0–0.0)	0.0 (0.0–0.0)	-
	T_1_	+16.7 (+12.5–+25.0)	+14.3 (+3.1–+23.8)	0.790
	T_2_	+114.0 (+80.0–+150.0)	+130.0 (+78.6–+261.0)	0.612
	T_3_	+100.0 (+66.7–+140.0)	+107.0 (+85.0–+184.0)	0.570
	T_4_	+75.0 (+50.0–+118.0)	+100.0 (+44.6–+148.0)	0.565
	T_5_	+44.4 (+25.0–+84.5)	+114.0 (+52.1–+190.0)	0.067
Hb (g dL^−1^)				
	T_0_	13.5 (12.5–14.3)	13.4 (12.5–14.8)	0.872
	T_1_	12.5 (11.5–13.0)	12.5 (11.6–13.8)	0.970
	T_2_	11.5 (10.8–12.0)	10.5 810.1–12.7)	1.000
	T_3_	12.0 (10.5–12.5)	11.5 (10.9–12.5)	0.814
	T_4_	12.0 (10.5–12.5)	10.5 (10.5–12.5)	1.000
	T_5_	11.8 (10.6–12.5)	10.6 (10.0–12.1)	0.648
Hb_Var_ (%)				
	T_0_	0.0 (0.0–0.0)	0.0 (0.0–0.0)	-
	T_1_	−6.7 (−8.2–−3.8)	−5.7 (−7.0–−4.3)	0.697
	T_2_	−13.8 (−16.7–−10.2)	−15.8 (−23.8–−12.7)	0.418
	T_3_	−11.5 (−16.5–−8.0)	−11.0 (−15.9–8.7)	0.923
	T_4_	−12.5 (−16.0–−7.5)	−13.3 (−21.3–−12.7)	0.347
	T_5_	−12.6 (−17.2–−8.3)	−16.7 (−25.4–−12.4)	0.310

The table reports median values (Q1–Q3) at six perioperative time points (T0 to T5), stratified by complications. Pairwise comparisons were performed with the Mann-Whitney test, and p-values were adjusted with a False Discovery Rate (FDR). An adjusted p-value< 0.05 was considered significant. SVI, Stroke Volume Index; SVI_Var_, Stroke Volume Index as percentage variations from baseline (T_0_); ScvO_2_ Central Venous Oxygen Saturation; ScvO_2Var_, Central Venous Oxygen Saturation as percentage variations from baseline (T_0_); SpO_2_, peripheral oxygen saturation; SpO_2Var_, peripheral oxygen saturation as percentage variations from baseline (T_0_); Lat_Var_, Lactate concentration as percentage variations from baseline (T_0_); Hb, Hemoglobin; Hb_Var_, Hemoglobin concentration as percentage variations from baseline (T_0_). T_0_: immediately after the induction of general anesthesia (start of PGDT protocol); T_1_: before aortic clamping; T_2_: after aortic declamping; T_3_: end of surgery; T_4_: two hours after admission to the POCU; T_5_: six hours after POCU admission (end of PGDT protocol).

## Discussion

This explorative retrospective analysis investigated postoperative outcomes in patients undergoing OEAAS within a structured PGDT protocol.

Brienza et al.^22^ in an accurate review and meta-analysis of the literature, demonstrated that adopting a PGDT protocol in patients considered to be at high risk was associated with a reduction in mortality rate compared to the control group. However, the strength of the recommendation is weak, with a moderate quality of evidence. The role of PGDT in vascular surgery, especially for open aortic procedures, is controversial, with conflicting literature. Initial studies showed no significant benefits of PGDT on morbidity or mortality.^12^, ^14^ For instance, Valentine et al.^13^ found no difference in postoperative morbidity, although the PGDT group had more intraoperative complications (18 % vs. 5 %, p = 0.02). Bisgaard et al.^15^ in a randomized trial, observed no change in postoperative complication rates with PGDT. Conversely, Funk et al.^16^ reported a significant reduction in complications with PGDT (12 vs. 28, p = 0.02), emphasizing the ongoing heterogeneity in outcomes and study designs.

Our cohort’s 30-day mortality rate was 4.0 %, consistent with reports for this surgical population (1–5 %) 2, 3, 4; however, among patients who developed postoperative complications, mortality reached 40.0 %, underscoring the impact of complications on short-term survival. Most deaths occurred in the context of postoperative complications, particularly mesenteric ischemia or bleeding. These observations align with robust evidence linking postoperative complications to both early and late mortality. Complications are a dominant driver of 30-day death and adversely affect long-term survival after major surgery.^23^

The overall complication rate was 9.9 %. V-POSSUM-adjusted bivariate regression did not reveal a single dominant predictor but rather a multifactorial interplay involving baseline patient risk, intraoperative complexity, and postoperative management. Within this framework, surgical duration emerged as a salient signal. Duration likely acts as a proxy for procedural strain, capturing technical difficulty and intraoperative challenges (e.g., bleeding, adhesions) or delays due to anatomical complexity. Longer operative time is associated with a higher risk of ischemic colitis and prolonged hospitalization^24^; in aortic surgery, complication profiles such as mesenteric/colonic ischemia are uncommon but carry very high mortality, particularly when operative time and intraoperative strain increase.^25^, ^26^ While not necessarily causal, prolonged operative time may extend exposure to physiological stressors (hypoperfusion, hemodilution/transfusion, inflammatory burden), thereby increasing the likelihood of postoperative complications and, consequently, early mortality.

The role of fluid balance, particularly in the postoperative period, emerged as a central factor in our cohort. A more positive fluid balance was significantly associated with the development of complications. This finding supports evidence that volume overload is detrimental, as excessive fluid administration has been linked to impaired pulmonary gas exchange, delayed gastrointestinal recovery, and renal dysfunction.^27^, ^28^ However, due to the study’s retrospective nature, a causal relationship cannot be established. On the one hand, it is plausible that fluid accumulation contributes directly to adverse outcomes by promoting tissue edema and impairing organ function. On the other hand, the need for ongoing fluid administration may reflect an underlying hemodynamic instability or organ dysfunction, making fluid overload a consequence rather than a cause of clinical deterioration. This behavior should alert the clinician: repeated fluid administration without clear benefit may signal an evolving complication rather than an isolated volume deficit. Before proceeding with additional fluid challenges, it is essential to reassess the clinical context and actively rule out complications responsible for persistent hemodynamic derangement (e.g., bleeding, infection, cardiac dysfunction, or capillary leak). Future studies should incorporate time-resolved fluid inputs/outputs and causal frameworks to clarify directionality.

In our cohort, NE bolus administration was inversely associated with postoperative complications. Notably, no patient experienced overt hypotension (per protocol definition), suggesting that complications may arise from factors beyond hypotension alone. Vasopressors are essential to maintain MAP and end-organ perfusion during major surgery,^29^, ^30^ and PGDT strategies that incorporate timely vasopressor use have been associated with fewer complications, shorter ICU/hospital stay, and improved recovery in selected surgical populations. However, this correlation warrants cautious interpretation: hemodynamic responses to vasopressors are patient-specific and context-dependent.^31^, ^32^ Within our PGDT pathway, NE boluses likely reflect proactive correction of transient hypotension rather than a truly protective vasopressor effect; confounding by indication and reverse causality cannot be excluded. Moreover, vasopressor exposure was recorded as bolus use rather than cumulative dose/timing, limiting causal inference. Future larger studies should capture time-resolved exposure (e.g., time-dependent covariates, MAP time-under-threshold, cumulative bolus dose/count) and adopt causal frameworks (e.g., marginal structural models), accounting for bleeding/transfusion, operative complexity, and anesthetic depth.

The temporal analysis of hemodynamic and ABG parameters in our cohort revealed clinically significant insights into the evolution of physiological status during and after open aortic surgery. Patients who developed complications showed significantly greater reductions in ScvO₂ during aortic clamping (T_1_) and at the end of surgery (T_3_), suggesting that episodes of impaired DO_2_ may have occurred despite protocol-driven optimization. In parallel, lactate levels measured at the end of the PGDT protocol (T_5_) were significantly higher in the complication group, pointing to persistent or unresolved tissue hypoperfusion.

These findings underscore the clinical value of dynamic physiological markers, particularly ScvO₂ and lactate, as sensitive indicators of the adequacy of perfusion and DO₂. ScvO₂ reflects the balance between oxygen delivery and consumption; a decline typically indicates insufficient DO₂ due to hypovolemia or reduced cardiac output, whereas an increase may suggest improved perfusion or reduced metabolic demand.^33^ Lactate, on the other hand, is a well-established marker of anaerobic metabolism and tissue hypoxia. Elevated intra- or postoperative lactate levels are frequently associated with inadequate perfusion and adverse outcomes. Importantly, not only absolute values but also temporal trends and clearance rates are prognostically relevant, particularly in the context of perioperative stress or circulatory compromise.^34^, ^35^ Our findings suggest that static single-point measurements may miss evolving physiological issues, while time-based trend analysis offers early insights into patient risk. To our knowledge, this is among the first studies to describe the temporal evolution of ScvO₂ and lactate within a structured PGDT protocol for OEAAS patients. This perspective provides a framework for refining individualized hemodynamic monitoring and postoperative management in high-risk vascular surgery.

This study has several limitations that must be acknowledged. First, PGDT aims to optimize perfusion and reduce complications; however, this study did not evaluate its effectiveness compared to standard care. Instead, it identified potential predictors of adverse outcomes in a cohort already managed with PGDT. Moreover, ASA IV patients or urgent/emergency procedures (e.g., ruptured/impending rupture), in which physiological reserve, time constraints, and hemodynamic goals may differ, were excluded. Extrapolation to these settings should therefore be cautious and requires dedicated validation. Second, the study was conducted in a single-center with a small sample size, particularly among patients who developed postoperative complications (n = 10); accordingly, it should be interpreted as exploratory and hypothesis-generating. Although we used Firth’s penalized likelihood method to mitigate small-sample bias in the logistic regression models, the low number of events may limit the generalizability of the findings and increase the risk of overfitting. Third, a complete multivariable regression analysis to identify independent risk factors was not feasible due to the low number of events. Likewise, small complication cases prevented threshold-based analyses to explore the predictive value of specific cut-off values for continuous physiological variables like lactate or ScvO₂. Fourth, our study examined hemodynamic and metabolic trends during multiple perioperative time points, but our observation was limited to the first six hours postoperatively. Consequently, some delayed physiological changes or complications may have gone undetected, potentially influencing interpretation. Fifth, several aspects of postoperative care that may confound outcomes were not systematically modeled. While we describe our standardized practices for patient warming, postoperative analgesia, and glycemic control, we did not collect time-resolved data (e.g., temperature curves, opioid dose/timing, glucose trends) and could not adjust for their effects; residual confounding is therefore possible and should be addressed prospectively in larger cohorts using time-resolved methods. Finally, although all patients followed a structured PGDT protocol and the same anesthesiologist attended all procedures, ensuring consistent decision-making, we did not formally assess protocol adherence or real-time achievement of hemodynamic targets. This limits our ability to determine if adverse outcomes resulted from protocol limitations or minor deviations in clinical application.

## Conclusions

This exploratory study identified key perioperative factors, such as prolonged surgical duration, postoperative fluid overload, altered ScvO₂ and lactate trends, associated with complications in patients undergoing OEAAS within a structured PGDT protocol. Despite protocol-guided optimization, adverse outcomes occurred and appeared linked to unresolved tissue hypoperfusion or evolving clinical deterioration. Further prospective studies are needed to confirm these associations and assess whether integrating dynamic perfusion markers into PGDT strategies can improve risk stratification and clinical outcomes in vascular surgery.

## CRediT authorship contribution statement

**Antonio Romanelli:** Writing – review & editing, Writing – original draft, Visualization, Validation, Software, Resources, Methodology, Investigation, Formal analysis, Data curation. **Rosanna Carmela De Rosa:** Writing – review & editing, Writing – original draft, Visualization, Validation, Supervision, Software, Resources, Project administration, Methodology, Investigation, Funding acquisition, Formal analysis, Data curation, Conceptualization. All authors have read and agreed to the published version of the manuscript.

## Consent for publication

Written informed consent has been obtained from the patients for this observational research.

## Ethical statement

The study was conducted by the Declaration of Helsinki, and approved by the local ethics committees (AORN dei Colli approval code: 794/2016).

## Funding

This research received no external funding.

## Data availability

The dataset used and analyzed during the current study is available from the corresponding author on reasonable request.

## Declaration of competing interest

The authors declare that they have no known competing financial interests or personal relationships that could have appeared to influence the work reported in this paper.
