# Supplementary material for: Perioperative predictors of complications in open abdominal aortic surgery: A retrospective analysis under a structured perioperative goal-directed therapy protocol
Source: J Anesth Transl Med. 2025 Dec 2;4(4):197–206. doi: 10.1016/j.jatmed.2025.10.002 (PMC13001745; doi:10.1016/j.jatmed.2025.10.002)
Supplement: Supplementary file 1 — Supplementary material [file mmc1.docx]

**Supplementary**

**Perioperative predictors of complications in open abdominal aortic surgery: a retrospective analysis under a structured perioperative goal-directed therapy protocol**

Rosanna Carmela De Rosa^a,*^, Antonio Romanelli^b^

^a^ Anesthesia and Intensive Care, AORN Ospedali dei Colli - "D. Cotugno" Hospital, Naples, Italy

^b^ Anesthesia and Intensive Care, AOU “San Giovanni di Dio e Ruggi d'Aragona”, Salerno, Italy

**Corresponding Author at:**

Rosanna Carmela De Rosa

Email: rosanna.derosa@ospedalideicolli.it

1. **Perioperative management**

***1.1 Intraoperative monitoring and anesthetic management***

Before entering the operating room, all patients were premedicated with morphine (10 mg, intramuscular).

Standard intraoperative monitoring included continuous electrocardiography (ECG), pulse oximetry (SpO₂), end-tidal carbon dioxide (EtCO₂), non-invasive blood pressure, and urine output. A large-bore peripheral venous catheter (16-14G) was inserted in the left arm, and either the radial or humeral artery was cannulated to allow continuous invasive blood pressure monitoring. Additional monitoring included depth of anesthesia via bispectral index (BIS™, Medtronic, Minneapolis, MN, USA), neuromuscular blockade monitoring (Tof Cuff^®^, RGB Medical Devices, Madrid, Spain), skin temperature, and continuous non-invasive hemoglobin measurement (Masimo Rainbow SET Radical-7^®^).

General anesthesia was induced with intravenous midazolam (0.07 mg/kg), propofol (0.7 mg/kg), sufentanil (0.4 mcg/kg), and rocuronium (0.6 mg/kg). Following orotracheal intubation, patients were mechanically ventilated (Drӓger Zeus^®^, Drӓger, Lubeck, Germany) in volume-controlled mode (tidal volume 6-8 ml/kg of ideal body weight, Auto Flow system, PEEP 5 cmH_2_O, respiratory rate 12-16/min keeping an EtCO_2_ between 35-40 mmHg, FiO_2_ 50%, closed circuit with automatic gas control).

Anesthesia maintenance included desflurane with a BIS target between 40 and 60, continuous remifentanil infusion (0.15 mcg/kg/min), and additional rocuronium (0.2 mg/kg) guided by neuromuscular monitoring.

After induction, a central venous catheter (PreSep™, Edwards Lifesciences, Irvine, CA, USA) was inserted via the internal jugular vein to enable continuous monitoring of central venous pressure (CVP) and central venous oxygen saturation (ScvO₂).

***1.2 Perioperative goal-directed therapy and fluid management protocol***

A structured PGDT protocol, based on stroke volume index (SVI) optimization, was implemented for all patients, beginning immediately after anesthesia induction and continuing for at least six hours postoperatively in the Postoperative Care Unit (POCU).

Hemodynamic monitoring was conducted using the EV1000™ clinical platform (Edwards Lifesciences, Irvine, CA, USA), employing pulse contour analysis via the FloTrac™ or VolumeView™ modules.

Following anesthesia induction, patients in stable hemodynamic conditions received a fluid challenge with a colloid solution (Gelofusine^®^, 4% modified fluid gelatin in saline, 3.0 ml/kg) within five minutes. The initial SVI was recorded, and the percentage change (ΔSVI%) was calculated after each fluid bolus. A response was defined as ΔSVI%>10%. In cases of a positive response, additional fluid challenges were administered every 10-15 minutes until the SVI increase following a bolus was ≤10%. The last SVI value associated with a positive response defined the individual’s maximal SVI (SVI_Max_). A personalized SVI trigger value was then calculated as follows:

SVI_Trigger_ = SVI_Max_ - 10%SVI_Max_

During surgery and in the early postoperative period, a new fluid challenge was administered whenever the patient’s SVI dropped below the SVI_Trigger_. If the mean arterial pressure (MAP) remained below 65 mmHg despite SVI optimization (e.g., after aortic declamping), intravenous norepinephrine (NE) boluses (0.01-0.02 mg) were administered to maintain adequate organ perfusion pressure.

Crystalloids (balanced electrolyte solution) were administered continuously at a 1.0-3.0 ml/kg/h basal rate. Intraoperative autologous blood salvage was performed whenever clinically appropriate. All fluids and blood products were warmed using a HOTLINE® fluid and blood warmer (37-39 °C) to prevent hypothermia.

At the end of the surgical procedure, patients were transferred to the POCU. Sedation was maintained with a continuous intravenous infusion of propofol and remifentanil, titrated according to hemodynamic stability and depth of sedation.

Standard monitoring in the POCU included continuous ECG, SpO₂, invasive arterial blood pressure, urine output, core temperature, CVP, and ScvO₂. Advanced hemodynamic monitoring was continued using the EV1000™ platform, and the same PGDT protocol initiated intraoperatively was applied during the first six postoperative hours. Active warming was provided to maintain normothermia (forced-air warming and warmed IV fluids).

As sedation was progressively reduced, the Richmond Agitation-Sedation Scale (RASS) was used to guide the transition to spontaneous ventilation. Once the patient exhibited adequate respiratory drive and gas exchange, pressure support ventilation was initiated, and spontaneous breathing trials were conducted. Extubation was performed upon successful trial completion and when all safety criteria were met.

During the initial 6-hour observation period, the remifentanil infusion was titrated to achieve NRS <3. In this phase, patients also received paracetamol 1 g IV every 8 hours and ketorolac 30 mg IV every 12 hours, as tolerated. At the end of the observation period, remifentanil was gradually tapered to discontinuation and overlapped with tramadol 100 mg IV every 12 hours as part of the multimodal regimen.

In accordance with our local protocol, peri-operative glycaemic control followed a stepwise approach: whenever blood glucose exceeded 180 mg/dL, 10 units of IV insulin were administered; if the subsequent measurement remained >180 mg/dL, an intravenous insulin infusion was commenced and titrated to achieve the target range.

**2. Aligned rank transformed (ART) test**

***2.1* *Mean Arterial Pressure***

The ART test for mean arterial pressure (MAP) showed a significant main effect of time (F(5, 495) = 18.99, p < 0.001), indicating substantial variation over time. There was a trend toward significance for the main effect of complication status (F(1, 99) = 3.19, p = 0.077), suggesting a possible difference in MAP between patients with and without complications. However, the interaction between time and complication was not significant (F(5, 495) = 0.58, p = 0.715), indicating that the temporal pattern of MAP did not differ substantially based on complication status.

For MAP, expressed as percentage variation (MAP_Var_) from T_0_ values, the ART test showed a significant main effect of time (F(5, 495) = 17.32, p < 0.001), indicating substantial variation over time. The main effect of complication status was not significant (F(1, 99) = 1.28, p = 0.261), suggesting no overall difference in MAP_Var_ between patients with and without complications. Similarly, the interaction between time and complication was not significant (F(5, 495) = 1.74, p = 0.125), indicating that the temporal pattern of MAP_Var_ did not significantly differ between the two groups.

***2.2* *Central Venous Pressure***

The ART test for central venous pressure (CVP) showed a significant main effect of time (F(5, 495) = 5.42, p < 0.001), indicating significant change over time. The main effect of complication status approached significance (F(1, 99) = 3.77, p = 0.055), suggesting a possible difference in CVP values between patients with and without complications. The interaction between time and complication was not significant (F(5, 495) = 1.03, p = 0.398), indicating similar temporal trends in CVP regardless of complication status.

For CVP, expressed as percentage variation (CVP_Var_), from T_0_ values, the ART test showed a significant main effect of time (F(5, 495) = 5.84, p < 0.001), indicating that CVP_Var_ varied significantly over time. No significant effect was found for complication status (F(1, 99) = 0.10, p = 0.752), nor was there a significant interaction between time and complication (F(5, 495) = 0.82, p = 0.533), suggesting that the temporal pattern of CVP_Var_ was similar between patients with and without complications.

***2.3* *Heart rate***

The ART test for heart rate (HR) showed a significant main effect of time (F(5, 495) = 24.86, p < 0.001), indicating substantial changes over time. The main effect of complication status approached significance (F(1, 99) = 2.73, p = 0.102), suggesting a possible difference in HR between patients with and without complications. However, the interaction between time and complication was not significant (F(5, 495) = 0.35, p = 0.884), indicating that the pattern of HR change over time was similar in both groups.

For HR, expressed as percentage variation (HR_Var_) from T_0_ values, the ART test showed a highly significant main effect of time (F(5, 495) = 20.30, p < 0.001). No significant main effect of complication status was found (F(1, 99) = 0.01, p = 0.919), nor was there a significant interaction between time and complication (F(5, 495) = 0.34, p = 0.888), suggesting that the temporal pattern of HR_Var_ did not differ between patients with and without complications.

***2.4* *Stroke Volume Index***

The ART test for stroke volume index (SVI) showed a significant main effect of time (F(5, 495) = 23.34, p < 0.001), indicating significant variations over time. No significant main effect was found for complication status (F(1, 99) = 1.22, p = 0.272). However, a significant interaction between time and complication was observed (F(5, 495) = 3.04, p = 0.010), suggesting that the temporal pattern of SVI differs between patients with and without complications.

For SVI, expressed as percentage variation (SVI_Var_) from T_0_ values, the ART test showed a highly significant main effect of time (F(5, 495) = 33.02, p < 0.001), indicating substantial variation in SVI_Var_ over time. The main effect of complication status was not significant (F(1, 99) = 1.36, p = 0.246). However, the interaction between time and complication was significant (F(5, 495) = 3.39, p = 0.005), suggesting that the temporal trajectory of SVI_Var_ differs between patients with and without complications.

***2.5* *Cardiac Index***

The ART test for cardiac index (CI) showed a significant main effect of time (F(5, 495) = 30.51, p < 0.001), indicating significant variation over time. No significant main effect of complication status was found (F(1, 99) = 0.21, p = 0.648). The interaction between time and complication approached significance (F(5, 495) = 2.13, p = 0.060), suggesting a possible differential trend in IC between patients with and without complications.

For CI, expressed as percentage variation (CI_Var_) from T_0_ values, the ART test showed a significant main effect of time (F(5, 495) = 39.52, p < 0.001), indicating substantial variation across time points. The main effect of complication status was not significant (F(1, 99) = 1.52, p = 0.221). However, the interaction between time and complication approached statistical significance (F(5, 495) = 2.11, p = 0.064), suggesting a possible difference in the temporal trajectory of CI_Var_ between groups.

***2.6* *ScvO_2_***

The ART test for ScvO_2_ showed a highly significant main effect of time (F(5, 495) = 64.23, p < 0.001), indicating significant variation over time. No significant main effect of complication status was observed (F(1, 99) = 0.45, p = 0.506). The interaction between time and complication approached statistical significance (F(5, 495) = 2.20, p = 0.053), suggesting a possible difference in the temporal pattern of ScvO₂ between patients with and without complications.

For ScvO₂, expressed as percentage variation (ScvO₂_Var_), the ART test showed a significant main effect of time (F(5, 495) = 61.98, p < 0.001), indicating dynamic changes over time.

A significant main effect of complication status was also observed (F(1, 99) = 6.62, p = 0.012), suggesting an overall difference in ScvO₂_Var_ response between patients with and without complications. A significant interaction between time and complication was found (F(5, 495) = 2.48, p = 0.031), indicating that the temporal profile of ScvO₂_Var_ differed between the two groups.

***2.7* *SpO_2_***

The ART test for SpO_2_ showed a highly significant main effect of time (F(5, 495) = 103.39, p < 0.001), indicating substantial variation across time points. A significant main effect of complication status was also observed (F(1, 99) = 10.87, p = 0.001), suggesting lower or altered SpO₂ levels in patients who experienced complications. Critically, the interaction between time and complication was highly significant (F(5, 495) = 8.45, p < 0.001), indicating that the temporal trajectory of SpO₂ differed markedly between groups.

For SpO₂, expressed as percentage variation (SpO₂_Var_), the ART test showed a highly significant main effect of time (F(5, 495) = 290.54, p < 0.001), reflecting substantial temporal dynamics.

The main effect of complication status was also highly significant (F(1, 99) = 11.87, p < 0.001), indicating that patients who developed complications exhibited different patterns of SpO₂_Var_. Furthermore, a highly significant interaction between time and complication status (F(5, 495) = 8.99, p < 0.001) revealed that the temporal evolution of SpO₂_Var_ markedly differed between groups.

***2.8* *pH***

The ART test for pH showed a significant main effect of time (F(5, 495) = 9.85, p < 0.001), indicating that pH varied significantly over time. No significant main effect of complication status (F(1, 99) = 0.43, p = 0.512) nor interaction between time and complication (F(5, 495) = 0.65, p = 0.664) were observed.

For pH, expressed as percentage variation (pH_Var_), the ART test showed a significant main effect of time (F(5, 495) = 8.94, p < 0.001), indicating that pH_Var_ varied significantly across time points. No significant effect of complication status (F(1, 99) = 0.22, p = 0.639) or time and complication interaction (F(5, 495) = 0.95, p = 0.448) was found, suggesting that pH_Var_ dynamics over time did not differ between groups.

***2.9* *Base Excess***

The ART test for base excess (BE) showed a significant main effect of time (F(5, 495) = 9.22, p < 0.001), indicating that BE varied significantly over time. However, no significant effect of complication status (F(1, 99) = 1.94, p = 0.167) or time and complication interaction (F(5, 495) = 0.96, p = 0.440) was found, suggesting that the BE trend over time was similar between groups.

***2.10* *Lactate***

The ART test for lactate showed a highly significant main effect of time (F(5, 495) = 58.77, p < 0.001), indicating that lactate concentrations varied substantially throughout the postoperative period. A significant main effect of complication status was found (F(1, 99) = 5.43, p = 0.022), suggesting that patients with complications had consistently different lactate levels. Critically, a highly significant interaction between time and complication status was identified (F(5, 495) = 13.82, p < 0.001), reflecting a distinct temporal lactate profile between patients with and without complications.

For lactate, expressed as percentage variation (Lat_Var_), the ART test showed a highly significant main effect of time (F(5, 495) = 60.03, p < 0.001), indicating substantial temporal changes in lactate dynamics. A significant main effect of complication status (F(1, 99) = 4.87, p = 0.030) showed that patients who experienced complications had different Lat_Var_ profiles. Furthermore, a highly significant time and complication interaction (F(5, 495) = 11.31, p < 0.001) confirmed that the trajectory of Lat_Var_ over time was markedly different between groups.

***2.11* *Hemoglobin***

The ART test for hemoglobin (Hb) showed a highly significant main effect of time (F(5, 495) = 37.71, p < 0.001), indicating substantial variation over time. No significant main effect of complication status was observed (F(1, 99) = 0.17, p = 0.685). However, a considerable time and complication interaction (F(5, 495) = 2.58, p = 0.026) revealed that the temporal pattern of Hb differed between groups.

For Hb, expressed as percentage variation (Hb_Var_), the ART test showed a highly significant main effect of time (F(5, 495) = 47.38, p < 0.001), indicating substantial variation across time points. A trend toward significance was found for the main effect of complication status (F(1, 99) = 3.67, p = 0.058), suggesting a possible overall difference in Hb_Var_ between groups. Notably, a significant interaction was observed between time and complication status (F(5, 495) = 3.66, p = 0.003), indicating that the temporal profile of Hb_Var_ differed between patients with and without complications.

| **Complication** | | **Incidence** |
| --- | --- | --- |
| **Respiratory** |  |  |
|  | Mechanical ventilation > 12 h | 2 (2.0 %) |
|  | Respiratory Failure | 2 (2.0 %) |
|  | Re-Intubation | 5 (5.0 %) |
| **Cardio-Vascular** |  |  |
|  | Hypotension treated with drugs | 1 (1.0%) |
|  | Ex-novo arrhytmia | 1 (1.0%) |
| **Renal** |  |  |
|  | Acute Renal Failure according to RIFLE/AKIN grading scheme | 6 (5.9%) |
| **Others** |  |  |
|  | Postoperative bleeding | 2 (2.0 %) |
|  | Mesenteric ischemia | 2 (2.0 %) |
|  | Intestinal Volvulus | 1 (1.0%) |
|  | Medullary Ischemia | 1 (1.0%) |

**Table S1: Organ-specific complications.**

Incidence is reported as absolute number and percentage (%).

RIFLE, Risk, Injury, Failure, Loss, End-stage kidney disease; AKIN, Acute Kidney Injury Network.

**Table S2: *Hemodynamic and arterial blood gas (ABG) analysis parameters presented as absolute values at each time point (T_0_–T_5_) and percentage changes relative to baseline (T_0_)*.**

| **Variable** | **Time** | **Absolute Values** | | **Percentage Variation** | |
| --- | --- | --- | --- | --- | --- |
|  |  | **Results** | **Min-Max** | **Results** | **Min-Max** |
| ***Mean Arterial Pressure (mmHg)*** | | |  |  |  |
|  | ***T_0_*** | 75.0 (70.0-80.0) | 60.0-95.0 | 0.0 (0.0-0.0) | 0.0-0.0 |
|  | ***T_1_*** | 75.0 (70.0-75.0) | 65.0-95.0 | -5.9 (-6.7-+7.1) | -19.4-+33.3 |
|  | ***T_2_*** | 75.0 (70.0-78.0) | 60.0-88.0 | 0.0 (-6.2-+6.7) | -20.0-+25.0 |
|  | ***T_3_*** | 80.0 (75.0-80.0) | 70.0-90.0 | +6.7 (0.0-+14.3) | -12.5-+33.3 |
|  | ***T_4_*** | 80.0 (77.0-80.0) | 65.0-90.0 | +6.7 (0.0-+14.3) | -19.4-+41.7 |
|  | ***T_5_*** | 80.0 (78.0-83.0) | 60.0-95.0 | +6.7 (0.0-+15.4) | -16.1-+41.7 |
| ***Central Venous Pressure (mmHg)*** | | |  |  |  |
|  | ***T_0_*** | 8.0 (7.0-9.0) | 4.0-13.0 | 0.0 (0.0-0.0) | 0.0-0.0 |
|  | ***T_1_*** | 8.0 (7.0-10.0) | 5.0-12.0 | 0.0 (-12.5-+14.3) | -28.6-+75.0 |
|  | ***T_2_*** | 7.0 (7.0-9.0) | 5.0-13.0 | 0.0 (-14.3-+16.7) | -37.5-+100.0 |
|  | ***T_3_*** | 8.0 (7.0-10.0) | 5.0-12.0 | 0.0 (-10.0-+14.3) | -25.0-+83.3 |
|  | ***T_4_*** | 7.0 (6.0-8.0) | 5.0-12.0 | 0.0 (-15.4-+11.1) | -37.5-+60.0 |
|  | ***T_5_*** | 7.0 (6.0-8.0) | 4.0-12.0 | -14.3 (-23.1-0.0) | -41.7-+75.0 |
| ***Heart Rate (bpm)*** | | |  |  |  |
|  | ***T_0_*** | 75.0 (70.0-75.0) | 55.0-90.0 | 0.0 (0.0-0.0) | 0.0-0.0 |
|  | ***T_1_*** | 73.0 (70.0-75.0) | 50.0-80.0 | -2.7 (-6.7-+4) | -20.0-+14.0 |
|  | ***T_2_*** | 73.0 (70.0-75.0) | 53.0-85.0 | -1.3 (-6.2-+4.0) | -22.7-+22.8 |
|  | ***T_3_*** | 75.0 (70.0-75.0) | 55.0-89.0 | 0.0 (-5.1-+2.7) | -14.3-+27.1 |
|  | ***T_4_*** | 78.0 (75.0-80.0) | 50.0-90.0 | +4.0 (0.0-+7.1) | -13.3-+31.6 |
|  | ***T_5_*** | 78.0 (77.0-80.0) | 57.0-100.0 | +6.2 (+2.7-+11.1) | -12.5-+42.9 |
| ***SVI (mL/min/m^2^)*** | | |  |  |  |
|  | ***T_0_*** | 35.0 (32.0-40.0) | 25.0-55.0 | 0.0 (0.0-0.0) | 0.0-0.0 |
|  | ***T_1_*** | 42.0 (38.0-45.0) | 27.0-56.0 | +20.0 (+10.0-+25.0) | -16.7-+50.0 |
|  | ***T_2_*** | 38.0 (35.0-42.0) | 25.0-54.0 | +6.7 (-1.8-+13.5) | -23.1-+44.8 |
|  | ***T_3_*** | 42.0 (40.0-45.0) | 30.0-57.0 | +17.1 (+8.6-+26.7) | -15.6-+55.6 |
|  | ***T_4_*** | 40.0 (37.0-43.0) | 30.0-53.0 | +14.0 (+5.7-+20.0) | -13.6-+50.0 |
|  | ***T_5_*** | 41.0 (38.0-45.0) | 28.0-56.0 | +15.6 (+8.6-+23.7) | -5.4-+48.2 |
| ***Cardiac Index (L/min/m^2^)*** | | |  |  |  |
|  | ***T_0_*** | 2.6 (2.3-2.8) | 1.9-3.8 | 0.0 (0.0-0.0) | 0.0-0.0 |
|  | ***T_1_*** | 2.9 (2.7-3.4) | 2.0-4.0 | +16.8 (+7.7-+22.9) | -21.9-+56.4 |
|  | ***T_2_*** | 2.8 (2.4-3.1) | 1.6-4.0 | +5.4 (-4.6-+12.6) | -25.9-+61.9 |
|  | ***T_3_*** | 3.1 (2.8-3.4) | 2.1-4.3 | +16.1 (+6.5-+25.0) | -15.6-+97.8 |
|  | ***T_4_*** | 3.1 (2.8-3.3) | 2.3-4.1 | +17.6 (+9.5-+27.0) | -13.6-+71.4 |
|  | ***T_5_*** | 3.2 (3.0-3.5) | 2.2-4.2 | +21.8 (+15.0-+31.7) | -2.7-+80.4 |
| ***ScvO_2_ (%)*** |  |  |  |  |  |
|  | ***T_0_*** | 83.0 (80.0-85.0) | 70.0-89.0 | 0.0 (0.0-0.0) | 0.0-0.0 |
|  | ***T_1_*** | 82.0 (78.0-83.0) | 72.0-87.0 | -2.3 (-2.5-+1.2) | -10.1-+9.3 |
|  | ***T_2_*** | 80.0 (78.0-83.0) | 73.0-88.0 | -2.3 (-3.5-0.0) | -12.8-+14.3 |
|  | ***T_3_*** | 80.0 (78.0-82.0) | 72.0-90.0 | -2.3 (-3.6-0.0) | -11.6-+15.7 |
|  | ***T_4_*** | 77.0 (75.0-79.0) | 70.0-85.0 | -6.0 (-8.2- -3.6) | -20.4-+7.1 |
|  | ***T_5_*** | 75.0 (74.0-77.0) | 70.0-82.0 | -8.2 (-10.0- -6.1) | -18.2-+5.7 |
| ***SpO_2_ (%)*** |  |  |  |  |  |
|  | ***T_0_*** | 100.0 (100.0-100.0) | 97.0-100.0 | 0.0 (0.0-0.0) | 0.0-0.0 |
|  | ***T_1_*** | 100.0 (100.0-100.0) | 98.0-100.0 | 0.0 (0.0-0.0) | -2.0-+3.1 |
|  | ***T_2_*** | 100.0 (100.0-100.0) | 97.0-100.0 | 0.0 (0.0-0.0) | -3.0-+3.1 |
|  | ***T_3_*** | 100.0 (100.0-100.0) | 97.0-100.0 | 0.0 (0.0-0.0) | -3.0-+2.1 |
|  | ***T_4_*** | 99.0 (98.0-100.0) | 95.0-100.0 | -1.0 (-1.0-0.0) | -5.0-0.0 |
|  | ***T_5_*** | 98.0 (97.0-98.0) | 96.0-100.0 | -2.0 (-3.0- -2.0) | -4.0-0.0 |
| ***pH*** |  |  |  |  |  |
|  | ***T_0_*** | 7.40 (7.39-7.42) | 7.31-7.47 | 0.0 (0.0-0.0) | 0.0-0.0 |
|  | ***T_1_*** | 7.40 (7.37-7.40) | 7.35-7.47 | -0.3 (-0.5-+0.3) | -0.9-+1.2 |
|  | ***T_2_*** | 7.37 (7.35-7.39) | 7.31-7.47 | -0.4 (-0.7- -0.1) | +1.7-+1.1 |
|  | ***T_3_*** | 7.40 (7.40-7.43) | 7.35-7.50 | +0.1 (-0.3-+0.4) | -0.9-+1.3 |
|  | ***T_4_*** | 7.39 (7.38-7.41) | 7.32-7.50 | -0.1 (-0.5-+0.3) | -1.1-+1.8 |
|  | ***T_5_*** | 7.40 (7.39-7.40) | 7.35-7.48 | -0.1 (-0.4-+0.1) | -1.1-+1.1 |
| ***Base Excess (mmol/L)*** | | |  |  |  |
|  | ***T_0_*** | 0.0 (-1.0-+2.0) | -3.0-+3.0 | 0.0 (0.0-0.0) | 0.0-0.0 |
|  | ***T_1_*** | 0.0 (-1.0-0.0) | -3.0-+2.3 | - | - |
|  | ***T_2_*** | -2.0 (-2.0- -1.0) | -5.0-+4.0 | - | - |
|  | ***T_3_*** | 0.0 (-1.0-+1.0) | -3.0-+4.0 | - | - |
|  | ***T_4_*** | -0.5 (-1.0-0.0) | -4.0-+3.0 | - | - |
|  | ***T_5_*** | 0.0 (-1.0-0.0) | -3.0-+3.0 | - | - |
| ***Lactate (mmol/L)*** | |  |  |  |  |
|  | ***T_0_*** | 0.7 (0.6-0.8) | 0.4-1.3 | 0.0 (0.0-0.0) | 0.0-0.0 |
|  | ***T_1_*** | 0.8 (0.7-1.0) | 0.5-1.7 | +16.7 (+12.5-+25.0) | -12.5-+183.0 |
|  | ***T_2_*** | 1.5 (1.2-1.7) | 0.8-11.0 | +114.0 (80.0-150.0) | +12.5-+1275.0 |
|  | ***T_3_*** | 1.5 (1.2-1.8) | 0.8-10.0 | +100.0 (+66.7-143.0) | 0.0-+1150.0 |
|  | ***T_4_*** | 1.2 (1.0-1.5) | 0.8-7.0 | +80.0 (+50.0-+122.0) | +11.1-+775.0 |
|  | ***T_5_*** | 1.0 (1.0-1.3) | 0.7-3.0 | +50.0 (+25.0-+87.5) | 0.0-+440.0 |
| ***Hemoglobin (g/dL)*** | |  |  |  |  |
|  | ***T_0_*** | 13.5 (12.5-14.5) | 8.1-16.5 | 0.0 (0.0-0.0) | 0.0-0.0 |
|  | ***T_1_*** | 12.5 (11.5-13.0) | 7.5-15.0 | -6.2 (-8.0- -3.8) | -32.1-+2.9 |
|  | ***T_2_*** | 11.5 (10.5-12.0) | 9.0-14.5 | -13.8 (-17.2- -10.3) | -36.2-+17.3 |
|  | ***T_3_*** | 12.0 (10.5-12.5) | 9.5-14.5 | -11.5 (-16.7- -8.0) | -27.6-+27.2 |
|  | ***T_4_*** | 11.8 (10.5-12.5) | 9.0-15.0 | -13.0 (-16.0- -7.7) | -36.2-+15.8 |
|  | ***T_5_*** | 11.5 (10.5-12.5) | 7.5-14.0 | -13.1 (-17.2- -8.6) | -42.3-+29.6 |

Descriptive statistics of key hemodynamic, metabolic, and respiratory parameters over time. Absolute values and percentage variations from baseline (T₀) are reported for each timepoint (T₁ to T₅) for the following variables: mean arterial pressure (MAP), central venous pressure (CVP), heart rate (HR), stroke volume index (SVI), cardiac index (CI), central venous oxygen saturation (ScvO₂), peripheral oxygen saturation (SpO₂), pH, base excess, lactate, and haemoglobin. Data are presented as median (Q_1_-Q_3_) and minimum–maximum range. T_0_: immediately after the induction of general anesthesia (start of PGDT protocol); T_1_: before aortic clamping; T_2_: after aortic declamping; T_3_: end of surgery; T_4_: two hours after admission to the POCU; T_5_: six hours after POCU admission (end of PGDT protocol).
